# Congenital Rhabdomyosarcoma: a different clinical presentation in two cases

**DOI:** 10.1186/s12887-018-1128-5

**Published:** 2018-05-15

**Authors:** Ida Russo, Virginia Di Paolo, Carmelo Gurnari, Angela Mastronuzzi, Francesca Del Bufalo, Pier Luigi Di Paolo, Angela Di Giannatale, Renata Boldrini, Giuseppe Maria Milano

**Affiliations:** 1 Department of Pediatric Hematology/Oncology, Bambino Gesù Children’s Hospital, IRCCS, Piazza di Sant’Onofrio 4, 00165 Rome, Italy; 2 Department of Radiology, Bambino Gesù Children’s Hospital, IRCCS, Piazza di Sant’Onofrio 4, 00165 Rome, Italy; 3 Department of Laboratories - Pathology Unit, Bambino Gesù Children’s Hospital, IRCCS, Rome, Italy. Piazza di Sant’Onofrio 4, 00165 Rome, Italy

**Keywords:** Rhabdomyosarcoma, Newborn, Rare disease

## Abstract

**Background:**

Rhabdomyosarcoma (RMS), one of the most common soft tissue sarcomas of childhood, is very rare in the neonatal period (0.4–2% of cases). In order to gain a deeper understanding of this disease at such age, patient and tumor features, as well as treatment modality and outcome need to be reported.

**Case presentation:**

We describe two cases with congenital RMS treated at Bambino Gesù Children’s Hospital between 2000 and 2016. They represent only 2.24% of all RMS patients diagnosed during that period in our Institution; this data is in agreement with the incidence reported in the literature. They reflect the two different clinical forms in which the disease may manifest itself. One patient, with the alveolar subtype (positive for specific PAX3-FOXO1 fusion transcript) and disseminated disease, had a fatal outcome with central nervous system (CNS) progression despite conventional and high dose chemotherapy. The other child, with the localized embryonal subtype, was treated successfully with conservative surgery and conventional chemotherapy, including prolonged maintenance therapy. He is disease free at 7 years of follow-up.

**Conclusions:**

RMS can also be diagnosed during the neonatal period. Given the young age, disease management is often challenging, and especially for the alveolar subtype, the outcome is dismal despite intensified multimodality therapy. In fact, it characteristically manifests with multiple subcutaneous nodules and progression most commonly occurs in the CNS (Rodriguez-Galindo et al., Cancer 92(6):1613–20, 2001). In this context, CNS prophylaxis could play a role in preventing leptomeningeal dissemination, and molecular studies can allow a deeper tumor characterization, treatment stratification and identification of new potential therapeutic targets.

## Background

Rhabdomyosarcoma (RMS) is one of the most common soft tissue sarcomas of childhood. In 5–10% of cases, it is diagnosed in children aged less than one year old, and may be congential in 0.4–2% [1–3]. Various reports indicate that, when the diagnosis occurs in the neonatal period, the prognosis is worse than in older children [2–7]. We retrospectively reviewed the medical records of 89 children affected by RMS treated between 2000 and 2016 at our Department and, among these, we found two congenital cases. We report the clinical, radiological and histological characteristics of these patients, as well as therapeutic approach and outcome, together with a literature review of congenital RMS.

## Case presentation

### Case #1

**Fig. 1:**
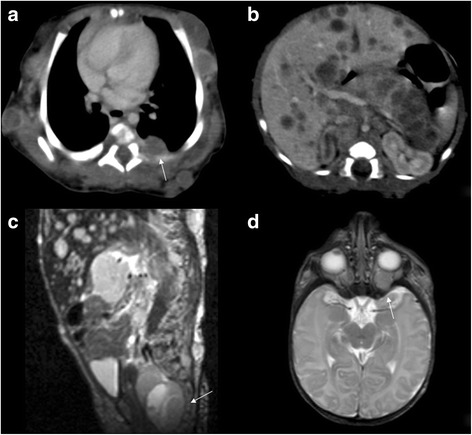
**a** Axial contrast-enhanced CT image shows a multiple soft tissue masses in the subcutaneous fat with peripheral enhancement; some of these masses expand in the muscles. In the left posterior part of the chest the mass can be seen to lie slightly lateral to the paravertebral region (white arrow). **b** Axial, contrast-enhanced CT shows multiple low-density masses in the right and left lobes of the liver and in the pancreas. **c** Sagittal T2-weighted MR imaging shows a lobulated, hyperintense presacral mass (white arrow). **d** Axial T2-weighted MR imaging shows heterogeneous hyperintense intraorbital mass (white arrow)

A full term newborn girl presented with widespread multiple nodular cutaneous and subcutaneous lesions on the head, limbs and trunk. Her mother’s pregnancy as well as family history were unremarkable. The color of the lesions ranged from bluish to purple, a characteristic of the so-called “blueberry muffin lesions”. She had no dysmorphic features or congenital anomalies. Excisional biopsy of one skin lesion showed a small-round-cell tumor, with an alveolar pattern of growth consistent with alveolar RMS (ARMS). Array-CGH analysis of tumor cells detected the specific PAX3-FOXO1 fusion transcript. Computed tomography (CT) revealed multiple lesions in the liver, pancreas, lungs, thoracic and abdominal walls (Fig. 1). A magnetic resonance image of the brain and spine showed a left retro-orbital mass and a paravertebral lumbosacral lesion. Bone-marrow and bones were positive for disease localization. After disease staging no primary site could be detected and the tumor was regarded as multifocal. The child was classified as group IV according to the Intergroup Rhabdomyosarcoma Study Group (IRSG) classification, and as stage IV according to TNM pretreatment staging classification [8]. She started chemotherapy with vincristine, actinomycin-D and cyclophosphamide (age and weight adapted doses). She presented a mixed response after 4 courses: complete remission in bone-marrow, skin and subcutaneous nodules; partial response of visceral, retro-orbital and lumbosacral lesions; leptomeningeal and pericardial progression. Following a discussion with the family and their request for further treatment, the child underwent chemotherapy with ifosfamide, vincristine, actinomycin-D, doxorubicin and intrathecal liposomial cytarabine arabinoside. The patient achieved complete remission of leptomeningeal and pericardial disease after 4 courses. Subsequently, she received high-dose consolidation therapy with busulfan and melphalan followed by autologous stem cell transplantation. Unfortunately, after one month, she presented central nervous system (CNS) recurrence and died of disease progression at 9 months of age.

### Case #2

A 5-day-old boy presented an abdominal mass in the hypogastric region. His mother’s full-term pregnancy was uncomplicated. Physical examination and routine neonatal laboratory values were normal. Ultrasound of the abdomen and pelvis showed a solid mass (greatest diameter was > 5 cm) located between the posterior bladder wall and sacrum. No family history of cancer was reported. On the assumption that the lesion was of benign nature, the child underwent macroscopically complete surgical excision of the mass. Intraoperatively, the tumor was found to originate from the prostate gland. The pathological examination revealed an embryonal RMS (ERMS). Molecular studies regarding the tumour with real time polymerase chain reaction (RT-PCR) did not reveal any PAX3-FOXO1 and PAX7-FOXO1 fusion transcripts. The child was classified as group II according to the IRSG classification and stage III according to the TNM pretreatment staging classification [8]. According to the European ongoing protocol (NCT#00339118) for older children, he started chemotherapy with 9 courses of vincristine, actinomycin-D and ifosfamide (age and weight adapted doses). Due to paralytic ileus, vincristine was reduced by 50% from the third course onwards. Considering the young age, radiotherapy was not delivered. Thus, it was decided to use a prolonged maintenance therapy with 6 cycles of cyclophosphamide and vinorelbine. He is disease free at 7 years of follow-up and is doing well except for mild neurogenic bladder dysfunction.

## Discussion and conclusion

Congenital RMS is a very rare neoplasia, as confirmed by data collected in our Institution over a 16-year period: only 2.24% of all RMS patients were diagnosed with the congenital form. We also performed a comprehensive PubMed search and 33 reports of congenital RMS (16 ERMS and 17 ARMS) published in the last 30 years were found and reviewed (Table 1). ERMS [9–22] ARMS [23–32]

**Table 1:** Congenital Rhabdomyosarcoma: review of the literature

Patient and disease characteristics	ERMS pts^e^ (*n* = 16)	ARMS pts^e^ (*n* = 17)
Gender: female/male (sex ratio)	5/11 (0.45)	10/7 (1.4)
Primary site		
PM head & neck	–	1
Non PM head & neck	6	4
Orbit	–	–
GU non bladder-prostate	2	–
GU bladder-prostate	2	–
Extremities	1	7
Other site	5^a^	4^b^
NA	–	1
IRSG		
I	1	–
II	4	1
III	11	3
IV	1	13^c^
Molecular biology		
Negative	2	5
NA	12	8
PAX3-FOXO1 positive	–	3
PAX7-FOXO1 positive	–	–
Others	2 [t(2;8)]	1 [N-myc amplification]
Therapy		
NA	–	1
Surgery only	2	–
Chemotherapy only	2	9
Chemotherapy + surgery	9	4
Chemotherapy + surgery + radiotherapy	3	2
Chemotherapy + radiotherapy	–	1
Outcome		
Alive [median months from dg (range)]	8 [31.5 (6–240)]	4 [9 (6–240)]
Dead	2	13^d^
NA	6	–

*ERMS* embryonal rhabdomyosarcoma, *ARMS* alveolar rhabdomyosarcoma, *pts.* patients, *PM* parameningeal, *n* number, *GU* genito-urinary, *NA* not available, *IRSG* Intergroup Rhabdomyosarcoma Study Group, *dg* diagnosis

^a^2-perineal, 2-chest wall, 1-trunk

^b^2-trunk, 2-chest wall

^c^For all patients, cutaneous and subcutaneous tissue was a metastatic site

^d^7/13 patients had central nervous system disease progression

^e^Data about family history of cancer were not available

The most frequent primary site for ARMS was the extremities, whereas for ERMS it was non-parameningeal head and neck. The two cases we reported represent the two different clinical forms in which the disease may manifest itself. While congenital ERMS is often localized and has the same behavior as that observed in older children, congenital ARMS is a highly malignant tumor, often occurring as a disseminated disease (see Case #1). Moreover, ARMS often evolves with the development of brain metastases despite an initial good response to chemotherapy [23, 24]. For case #1, in order to obtain CNS disease remission, we used intrathecal liposomial cytarabine arabinoside, and given the well-known dismal prognosis of the disease and the absence of other suitable alternatives, we decided to use consolidation high-dose chemotherapy. However, the results with high-dose chemotherapy reported in previous published trials, did not show significant benefits in metastatic RMS [33, 34]. Currently, there are no specific guidelines regarding treatment for neonates and infants with sarcoma, with few exceptions, such as that of infantile fibrosarcoma [35]. Infants with RMS are usually treated according to the same protocols used for older children: mainly alkylating agents, vincristine, actinomycin-D, with or without anthracyclines. However, they require tailored treatments given the physiologic immaturity of various organs. Ragab et al. [2] reported an unacceptable toxicity compared with results in older children (5% versus 1% of treatment-related deaths), when full chemotherapy doses were used in infants treated in the IRSG I and II trials. Chemotherapy dose reduction in infants resulted in less fatal toxicities without affecting the overall outcome [1, 3, 4]. Moreover, to avoid cardiac and renal damage, anthracyclines and ifosfamide are omitted in patients less than 3 and 1 months old respectively. With regards to the management of congenital ARMS, we hypothesized that children affected by this disease, could benefit from early use of chemotherapeutic agents with good blood-brain barrier passage as well as early intrathecal chemotherapy, as one of the main causes of treatment failure is CNS progression. Local control, determined by extended surgery and radiotherapy, also poses special challenges in very young children due to possible sequelae. In the literature (Table 1), only 3 out of 16 patients with congenital ERMS received radiotherapy associated with conservative surgery (one of them was a female with a vaginal primary who underwent brachytherapy) [23]. Given the small number of patients and the lack of follow-up data in about one third of cases (6\16), no conclusion can be drawn concerning the outcome. The Italian Cooperative Group reported a higher local recurrence rate in infants with RMS who did not receive appropriate local treatment [1]. In our ERMS patient, since radiotherapy was not recommended, we decided to prolong treatment with maintenance chemotherapy. In this context, our decision was taken in order to consolidate disease remission. The potential role of maintenance therapy in this setting is intriguing but impossible to define given the anecdotal nature of our case. Few data are available regarding tumor biology and fusion status, being reported in only 13 out of 33 cases (Table 1). Gene profiling, currently mandatory in RMS, is even more important in congenital forms, which present a challenging disease. In fact, it could allow more accurate prognostic predictions, as well as detection of new molecular targets. In this regard, molecular prognostic factors have already been identified for a subgroup of congenital RMS, namely the spindle-cell type [36]; the tumors carrying NCOA2 gene rearrangements indeed showed a more favorable clinical course.

In conclusion, although rarely, RMS can also arise in the neonatal period. In these patients, it is often difficult to establish a balance between the necessity to cure and the risk of long-term effects. A large effort to elaborate guidelines/protocols is desirable to homogenize treatment for this rare tumor occurring within this age group. From experience gathered in the 2 reported cases, early CNS prophylaxis should be considered for the alveolar subtype and prolonged maintenance chemotherapy rather than radiotherapy might be envisaged for the localized embryonal type. Moreover, deeper molecular biology studies are crucial for tumor characterization, treatment stratification and for the discovery of new therapeutic targets.

## Abbreviations

AIEOP: Italian Association of Pediatric Hematology/Oncology
ARMS: Alveolar Rhabdomyosarcoma
Array-CGH: Array - Comparative Genomic Hybridization
CT: Computed tomography
ERMS: Embryonal Rhabdomyosarcoma
IRSG: Intergroup Rhabdomyosarcoma Study Group
RMS: Rhabdomyosarcoma
